# Development and validation of AI models using LR and LightGBM for predicting distant metastasis in breast cancer: a dual-center study

**DOI:** 10.3389/fonc.2024.1409273

**Published:** 2024-06-14

**Authors:** Wen-hai Zhang, Yang Tan, Zhen Huang, Qi-xing Tan, Yue-mei Zhang, Bin-jie Chen, Chang-yuan Wei

**Affiliations:** ^1^ Department of Breast Surgery, Guangxi Medical University Cancer Hospital, Nanning, Guangxi, China; ^2^ Department of Pathology, The First Affiliated Hospital of Guangxi Medical University, Nanning, Guangxi, China

**Keywords:** distant metastasis, breast cancer, AI, LR, LightGBM algorithm

## Abstract

**Objective:**

This study aims to develop an artificial intelligence model utilizing clinical blood markers, ultrasound data, and breast biopsy pathological information to predict the distant metastasis in breast cancer patients.

**Methods:**

Data from two medical centers were utilized, Clinical blood markers, ultrasound data, and breast biopsy pathological information were separately extracted and selected. Feature dimensionality reduction was performed using Spearman correlation and LASSO regression. Predictive models were constructed using LR and LightGBM machine learning algorithms and validated on internal and external validation sets. Feature correlation analysis was conducted for both models.

**Results:**

The LR model achieved AUC values of 0.892, 0.816, and 0.817 for the training, internal validation, and external validation cohorts, respectively. The LightGBM model achieved AUC values of 0.971, 0.861, and 0.890 for the same cohorts, respectively. Clinical decision curve analysis showed a superior net benefit of the LightGBM model over the LR model in predicting distant metastasis in breast cancer. Key features identified included creatine kinase isoenzyme (CK-MB) and alpha-hydroxybutyrate dehydrogenase.

**Conclusion:**

This study developed an artificial intelligence model using clinical blood markers, ultrasound data, and pathological information to identify distant metastasis in breast cancer patients. The LightGBM model demonstrated superior predictive accuracy and clinical applicability, suggesting it as a promising tool for early diagnosis of distant metastasis in breast cancer.

## Background

Breast cancer is one of the most common malignancies affecting women worldwide, posing a significant threat to women’s health. By 2020, breast cancer had become one of the most frequently diagnosed cancers globally (1). While ranking fourth in terms of mortality, it showed the most significant increase in new death cases (1). In China alone, there are over 410,000 new cases of breast cancer annually, with over 110,000 associated deaths (2). The majority of deaths result from cancer metastasis, with approximately 20–30% of breast cancer patients likely to experience this occurrence (3).

Distant metastasis is a common form of recurrence and a lifelong risk for breast cancer patients (4). The sites of breast cancer metastasis are closely linked to patient survival, with the most common sites being bones, lungs, and liver (3, 5). Distant metastasis significantly diminishes the quality of life for breast cancer patients and can lead to mortality (4, 6).

Before the pathological confirmation of breast cancer metastasis through biopsy, MRI and CT scans are usually conducted to provide relevant indications (7, 8). When results from these imaging studies are inconclusive, diagnostic information may be provided by functional imaging modalities such as positron emission tomography, dynamic contrast-enhanced magnetic resonance imaging, or diffusion-weighted magnetic resonance imaging (9). The decision to conduct a series of imaging examinations for breast cancer patients entirely depends on the clinical suspicion of the physician, and even in cases of suboptimal results, expensive functional imaging studies may be required. For patients in some developing countries, the cost of multiple imaging examinations can be relatively high, resulting in a significant economic burden. Additionally, imaging examinations have certain limitations in certain situations (7).

To address these challenges, some studies have begun to explore the use of artificial intelligence (AI) technology to assist in predicting breast cancer metastasis (10–15). This AI-based approach holds promise for providing faster and more accurate diagnoses, while potentially reducing the need for expensive imaging studies, thereby alleviating the economic burden on patients. The current research primarily focuses on predicting the risk of breast cancer metastasis in the future (1 year, 3 years, or 5 years) (10, 12, 13, 15–18), while there is relatively less emphasis on diagnostic predictions for distant metastasis of breast cancer (11, 14, 19–21). In the study by Huang et al., the SEER database was used to predict bone metastasis in invasive ductal carcinoma; however, their study did not mention a validation set (11). Ma et al. developed a fusion model integrating clinical-pathological data with MRI features, which also showed promising performance (14). Similarly, Li et al. (19) also utilized MRI features and clinical pathological characteristics to establish a predictive model, but they did not mention the machine learning algorithms used, nor did they validate the model with external data. Additionally, Zhao et al. (20) used the SEER database and four machine learning algorithms, including Extreme Gradient Boosting (XGBoost), k-Nearest Neighbors (KNN), Decision Tree (DT), and Support Vector Machine (SVM) to predict the risk of distant metastasis in breast cancer, with XGBoost performing the best. Furthermore, Burak Yagin et al. (21) used genomic data from 98 breast cancer cases and several algorithms including Light Gradient Boosting Machine (LightGBM), Categorical Boosting (CatBoost), Extreme Gradient Boosting (XGBoost), Gradient Boosted Trees (GBT), and Adaptive Boosting (AdaBoost) to build a model for predicting distant metastasis in breast cancer, with LightGBM being the best performer. The above studies suggest that using AI to evaluate breast cancer metastasis before conducting relatively expensive whole-body imaging studies may help eliminate unnecessary imaging examinations.

In this study, we established AI models to identify breast cancer metastasis by integrating clinical blood markers, ultrasound data, and breast biopsy pathology. The algorithms used include not only the well-performing XGBoost and LightGBM from previous research but also AdaBoost and Logistic Regression (LR). This method not only improves the affordability and accessibility of diagnosis but also offers new avenues and possibilities for the early diagnosis of breast cancer metastasis. With ongoing technological advancements and deeper research, the application of AI in predicting breast cancer metastasis holds promise as a significant future development direction.

## Materials and methods

### Patient population

**Figure 1:**
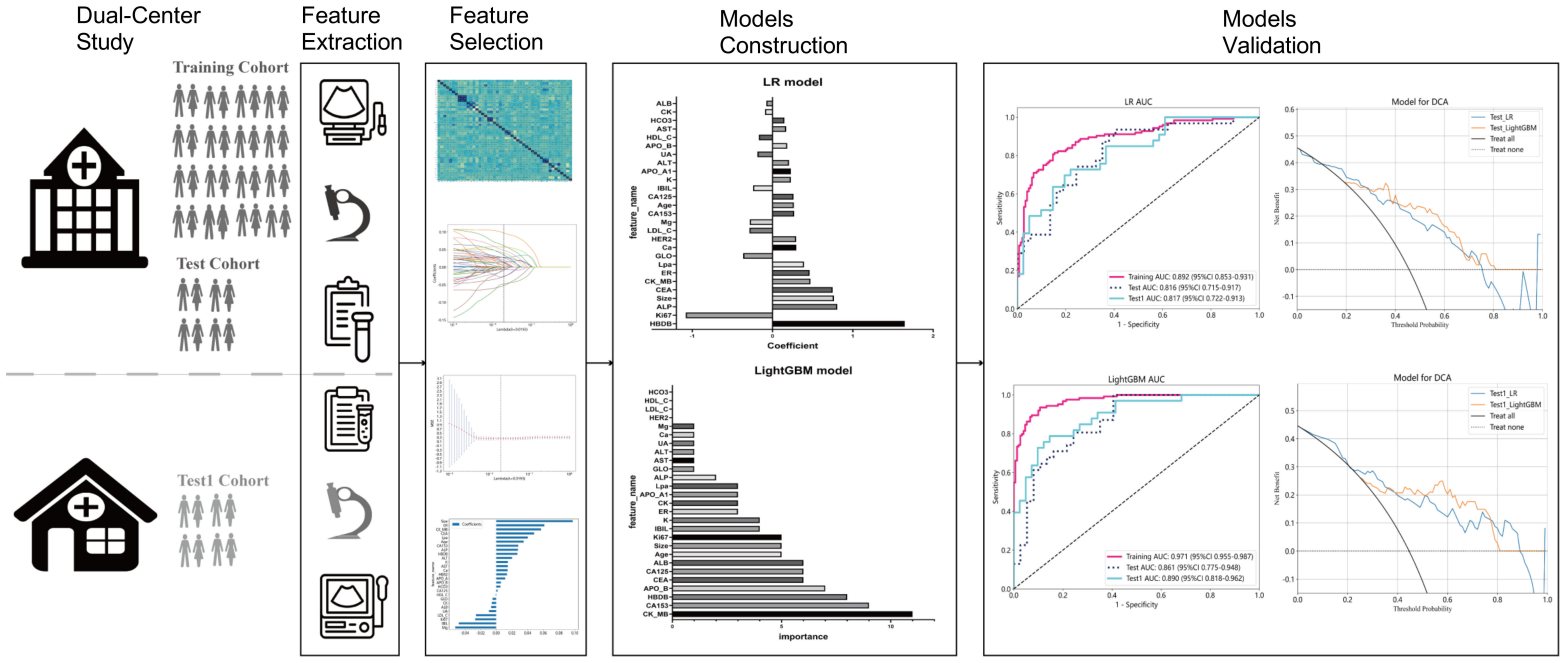
The workflow of LR and LightGBM models in this study.

This retrospective study included data from two medical centers, approved by the institutional review boards of both centers. Inclusion criteria were as follows: (1) definitive diagnosis of *de novo* primary breast cancer with or without distant metastasis; (2) completion of ultrasound examination, clinical blood marker testing, and breast biopsy pathology examination before treatment (radiotherapy or chemotherapy) or surgical resection; (3) no history of hypertension, diabetes, or hyperlipidemia; (4) no history of abnormalities in liver, kidney, or cardiovascular function blood markers; (5) no history of other diseases. Exclusion criteria were as follows: (1) distant metastasis occurred after treatment (surgical resection or chemotherapy); (2) ultrasound examination not performed due to unavoidable reasons (such as breast surface dressing coverage); (3) ultrasound examination did not provide the maximum diameter of the lesion; (4) clinical blood markers did not include tumor markers (AFP, CEA, CA125, CA153, and CA199), liver function tests, kidney function tests, lipid profile, or cardiovascular function markers; (5) the biopsy pathology examination did not provide immunohistochemical results for ER, PR, HER2, or Ki67. The breast cancer cases involved in the study were from two research centers, one comprising 342 patients randomly divided into training (274 patients) and test (68 patients) cohorts at an 8:2 ratio, and the other center’s 75 patients served as an external testing set (test1 cohort). Given that breast cancer distant metastasis in this study mainly occurs in the bones, lungs, and liver, with detailed local distributions outlined in **Table 1**. The workflow of the study’s model is illustrated in **Figure 1**.

**Table 1:** Clinical blood markers, pathological, and ultrasound characteristics in the training, test, and test1 cohorts.

Characteristics	Training cohort (n = 274)	Test cohort (n = 68)	Test1 cohort (n = 75)	*P* value
Age (years), mean ± SD	49.54 ± 9.96	48.91 ± 11.75	50.72 ± 10.69	0.563
Weight (kg), mean ± SD	57.44 ± 8.16	57.34 ± 9.07	56.61 ± 8.94	0.753
Maximum tumor diameter by ultrasound (cm), median (IQR)	3.70 (2.80, 5.00)	3.60 (2.45, 4.87)	2.60 (2.00, 4.50)	<0.001
CEA (ng/ml), median (IQR)	2.25 (1.34, 4.63)	2.62 (1.62, 5.19)	2.58 (1.48, 5.06)	0.296
AFP (ng/ml), median (IQR)	2.51 (1.80, 4.38)	2.52 (1.91, 3.41)	2.41 (1.79, 3.40)	0.728
CA125 (U/ml), median (IQR)	16.20 (10.84, 28.25)	15.70 (10.60, 22.58)	16.35 (10.18, 30.00)	0.858
CA153 (U/ml), median (IQR)	16.00 (9.75, 30.30)	15.15 (11.08, 31.15)	18.35 (9.18, 46.15)	0.833
CA199 (U/ml), median (IQR)	8.80 (3.75, 20.35)	9.75 (3.95, 19.75)	9.56 (3.09, 20.69)	0.686
TBIL (μ mol/L), median (IQR)	11.60 (8.95, 14.70)	11.00 (9.70, 13.00)	12.10 (10.10, 16.58)	0.172
DBIL (μ mol/L), median (IQR)	3.50 (2.80, 4.90)	3.65 (2.90, 4.38)	3.15 (2.30, 4.43)	0.106
IBIL (μ mol/L), median (IQR)	7.70 (6.10, 9.95)	7.50 (6.40, 8.93)	9.25 (7.20, 12.90)	0.004
TP (g/L), median (IQR)	69.70 (65.35, 74.00)	69.55 (66.85, 72.30)	71.30 (67.45, 75.78)	0.079
ALB (g/L), median (IQR)	40.00 (37.40, 43.05)	40.05 (38.20, 41.78)	39.25 (36.60, 41.38)	0.108
GLO (g/L), median (IQR)	29.30 (26.65, 31.95)	29.85 (26.65, 31.68)	33.25 (28.73, 36.10)	<0.001
A_G (Ratio), median (IQR)	1.37 (1.23, 1.53)	1.38 (1.21, 1.58)	1.20 (1.00, 1.40)	<0.001
GGT (U/L), median (IQR)	19.00 (16.00, 27.00)	17.50 (14.00, 26.00)	21.50 (13.00, 32.25)	0.438
TBA (μ mol/L), median (IQR)	3.90 (2.20, 6.65)	2.70 (1.80, 4.35)	4.75 (2.98, 7.60)	<0.001
PA (mg/L), median (IQR)	232.90 (201.20, 271.80)	236.00 (203.90, 262.88)	245.30 (201.95, 272.18)	0.885
AST (U/L), median (IQR)	25.00 (20.00, 31.00)	26.00 (20.00, 30.50)	23.50 (18.00, 32.25)	0.474
ALT (U/L), median (IQR)	15.00 (11.00, 22.00)	14.50 (11.00, 22.50)	14.50 (12.00, 19.25)	0.794
AST_ALT (Ratio), median (IQR)	1.62 (1.23, 2.01)	1.55 (1.23, 2.29)	1.65 (1.28, 2.15)	0.904
ALP (U/L), median (IQR)	68.00 (51.00, 86.00)	65.00 (50.25, 88.75)	77.50 (52.00, 96.25)	0.467
CHE (U/L), mean ± SD	8725.57 ± 2262.12	8507.16 ± 2116.71	8422.32 ± 2072.70	0.503
UREA (mmol/L), median (IQR)	4.50 (3.70, 5,50)	4.40 (3.83, 5.00)	4.42 (3.57, 5.24)	0.793
CREA (μ mol/L), median (IQR)	60.00 (53.00, 67.00)	58.00 (52.25, 67.00)	59.00 (51.00, 68.00)	0.960
UA (μ mol/L), median (IQR)	310.00 (252.00, 358.00)	290.50 (240.75, 363.25)	294.00 (253.25, 360.75)	0.688
HCO3 (mmol/L), median (IQR)	26.00 (24.00, 27.00)	26.00 (24.00, 28.00)	25.25 (22.98, 26.58)	0.042
Ccr (ml/min), mean ± SD	94.31 ± 24.98	96.97 ± 25.42	98.24 ± 21.09	0.403
CYSC (mg/L), median (IQR)	0.82 (0.71, 0.95)	0.80 (0.69, 0.90)	0.78 (0.69, 0.91)	0.209
K (mmol/L), median (IQR)	4.00 (3.70, 4.20)	3.90 (3.70, 4.18)	3.96 (3.73, 4.18)	0.422
Na (mmol/L), median (IQR)	141.00 (139.00, 142.00)	141.00 (140.00, 143.00)	139.85 (137.86, 141.00)	<0.001
Cl (mmol/L), median (IQR)	102.00 (101.00, 104.00)	103.00 (101.00, 104.00)	105.50 (104.08, 107.23)	<0.001
Ca (mmol/L), median (IQR)	2.29 (2.20, 2.38)	2.28 (2.20, 2.35)	2.29 (2.22, 2.36)	0.765
Mg (mmol/L), median (IQR)	1.00 (0.91,1.08)	1.03 (0.94, 1.09)	0.87 (0.82, 0.91)	<0.001
PHO (mmol/L), median (IQR)	1.08 (1.00, 1.20)	1.08 (1.00, 1.17)	1.12 (1.05, 1.21)	0.219
CK (U/L), median (IQR)	71.00 (58.00, 94.00)	74.00 (61.00, 97.00)	71.00 (52.00, 95.00)	0.457
CK_MB (U/L), median (IQR)	11.00 (8.00, 16.00)	12.00 (8.25, 14.00)	14.00 (11.00, 19.00)	0.001
LDH (U/L), median (IQR)	178.00 (153.50, 210.00)	174.00 (153.00, 203.00)	163.00 (141.50, 207.50)	0.226
HBDB (U/L), median (IQR)	134.00 (117.00, 159.00)	130.50 (114.50, 155.75)	123.00 (103.50, 145.50)	0.023
CHO (mmol/L), median (IQR)	4.97 (4.38, 5.79)	4.80 (4.38, 5.58)	4.98 (4.44, 5.87)	0.604
TG (mmol/L), median (IQR)	1.08 (0.77, 1.60)	1.11 (0.86, 1.70)	1.24 (0.81, 1.74)	0.398
HDL_C (mmol/L), median (IQR)	1.34 (1.15, 1.58)	1.25 (1.12, 1.46)	1.36 (1.18, 1.51)	0.159
LDL_C (mmol/L), median (IQR)	2.94 (2.47, 3.58)	2.91 (2.38, 3.42)	3.02 (2.51, 3.81)	0.522
APO_A1 (g/L), median (IQR)	1.31 (1.16, 1.46)	1.24 (1.15, 1.40)	1.30 (1.18, 1.46)	0.409
APO_B (g/L), median (IQR)	0.92 (0.77, 1.10)	0.92 (0.80, 1.13)	0.88 (0.73, 1.06)	0.159
A1_B1 (Ratio), median (IQR)	1.42 (1.18, 1.71)	1.38 (1.17, 1.57)	1.50 (1.20, 1.80)	0.324
Lpa (mg/L), median (IQR)	188.00 (98.50, 395.50)	139.50 (90.25, 300.00)	166.00 (118.25, 355.75)	0.447
Pathological diagnosis*, n (%)				0.872
Infiltrating duct carcinoma, NOS	258(94.16)	65(95.59)	72(97.30)	
Infiltrating lobular carcinoma, NOS	7(2.55)	3(4.41)	1 (1.35)	
Invasive papillary carcinoma	1(0.36)	0(0.00)	0(0.00)	
Invasive micropapillary carcinoma	4(1.46)	0(0.00)	0(0.00)	
Mucinous adenocarcinoma	3(1.09)	0(0.00)	1(1.35)	
Metaplastic carcinoma	1(0.36)	0(0.00)	0(0.00)	
Distant metastasis*, n (%)				0.803
None metastasis	150(54.74)	37(54.41)	41(55.41)	
Bone metastasis	65(23.72)	21(30.88)	17(22.97)	
Lung metastasis	35(12.77)	6(8.82)	8(10.81)	
Liver metastasis	24(8.76)	4(5.88)	8(10.81)	
Lymph node metastasis*, n (%)				0.350
Absent	114(41.61)	24(35.29)	35(47.30)	
Present	160(58.39)	44(64.71)	39(52.70)	
ER (Positive ratio), n (%)				0.403
≤ 0.5	134(48.91)	39(57.35)	35(47.30)	
>0.5	140(51.09)	29(42.65)	39(52.70)	
PR (Positive ratio), n (%)				0.300
≤ 0.1	146(53.28)	40(58.82)	34(45.95)	
>0.1	128(46.72)	28(41.18)	40(54.05)	
HER2(IHC), n (%)				0.119
0	72(26.28)	14(20.59)	25(33.78)	
1+	30(10.95)	9(13.24)	15(20.27)	
2+	79(28.83)	19(27.94)	13(17.57)	
3+	93(33.94)	26(38.24)	21(28.38)	
Ki67 (Positive ratio), n (%)				0.298
≤ 0.4	154(56.20)	39(57.35)	49(66.22)	
>0.4	120(43.80)	29(42.65)	25(33.78)	

SD, Standard deviation; AFP, Alpha-Fetoprotein; CEA, Carcinoembryonic Antigen; CA125, Carbohydrate Antigen125; CA153, Carbohydrate Antigen153; CA199, Carbohydrate Antigen 199; TBIL, Total bilirubin; DBIL, Direct bilirubin; IBIL, Indirect bilirubin; TP, Total protein; ALB, Albumin; GLO, globulin; A_G (Ratio), Albumin-globulin ratio; GGT, γ-glutamyl transferase; TBA, Total bile acids; PA, Pre-albumin; AST, Aspartate aminotransferase; ALT, Alanine aminotransferase; AST_ALT (Ratio), Aspartate aminotransferase to alanine aminotransferase ratio; ALP, Alkaline phosphatase; CHE, Cholinesterase; UREA, Urea; CREA, Creatinine; UA, Uric acid; HCO3, Blood bicarbonate concentration; Ccr, endogenous creatinine clearance rate; CYSC, Cysteine Protease Inhibitor C; K, Potassium ion; Na, Sodium ion; Cl, chloride ion; Ca, Calcium ion; Mg, Magnesium ion; PHO, Inorganic phosphate; CK, Creatine kinase; CK_MB, Creatine kinase isoenzyme; LDH, Lactate dehydrogenase; HBDB, α-hydroxybutyrate dehydrogenase; CHO, Total cholesterol; TG, Triglycerides; HDL_C, High-density lipoprotein cholesterol; LDL_C, Low-density lipoprotein cholesterol; APO_A1, Apolipoprotein A1; APO_B, Apolipoprotein B; A1_B (Ratio), Apolipoprotein A1 to apolipoprotein B ratio; Lpa, lipoprotein (a).

*, indicates that this characteristic does not need to be included in model construction.

### Feature extraction and selection

Features extracted from clinical blood markers included tumor markers (carcinoembryonic antigen, alpha-fetoprotein, CA125, CA153, and CA199), liver function indicators (total bilirubin, direct bilirubin, indirect bilirubin, total protein, albumin, globulin, albumin-globulin ratio, γ-glutamyl transferase, pre-albumin, aspartate transaminase (AST), alanine transaminase (ALT), AST/ALT ratio, alkaline phosphatase, cholinesterase, and total bile acids), kidney function indicators (urea, creatinine, uric acid, blood bicarbonate concentration, endogenous creatinine clearance rate, Cysteine Protease Inhibitor C, potassium ion, sodium ion, chloride ion (Cl), calcium ion, Magnesium ion (Mg), and inorganic phosphorus), lipid profile [total cholesterol, triglycerides, high-density lipoprotein cholesterol, low-density lipoprotein cholesterol, apolipoprotein A1, apolipoprotein B, A1/B ratio, and lipoprotein (a)], and cardiovascular function indicators [creatine kinase, creatine kinase isoenzyme (CK-MB), lactate dehydrogenase, and alpha-hydroxybutyrate dehydrogenase (α-HBDH)]. Features from ultrasound data included the maximum diameter of breast cancer lesions. Pathological data included immunohistochemical results for ER, PR, HER2, and Ki67.

The extracted features underwent the following procedures: first, standardization was performed using z-score normalization (mean = 0, standard deviation = 1) to preprocess the data to conform to a standard normal distribution. Next, Spearman rank correlation coefficient was utilized for statistical analysis to measure the correlation between two variables. When the Spearman correlation coefficient between features was >0.9, one of the highly correlated features was retained. This method employs a “greedy approach”. It selects the most redundant feature at each step to retain, aiming to minimize the correlation between features and thus enhance the models’ generalization ability and performance. Finally, LASSO regression with L1 regularization was employed for feature dimensionality reduction. This method selects highly correlated features and generates sparse models, meaning only a few features significantly contribute to the prediction results, thereby improving the interpretability and generalization capability of the model.

### Development and validation of models

LR, LightGBM, GBoost and AdaBoost machine learning algorithms were employed in this study to construct models for breast cancer with and without distant metastasis as binary outcome variables. Model construction was performed based on 5-fold cross-validation of the training set. After model construction, validation was conducted on the internal and external testing sets. Performance evaluation was conducted using metrics such as the area under the receiver operating characteristic curve (AUC), accuracy, sensitivity, specificity, positive predictive value (PPV), and negative predictive value (NPV). Subsequently, clinical decision curve analysis (DCA) was performed, reflecting the net benefit of different threshold probabilities in the training and internal and external validation sets to assess the clinical efficiency of the model.

### Statistical analysis

The analysis of clinical baseline features was performed using SPSS software (version 25.0, IBM). For the comparison of normally distributed continuous variables with homogeneity of variance (expressed as x ± s) across multiple groups, ANOVA was used. For the comparison of non-normally distributed or heteroscedastic continuous variables (expressed as median (IQR)) across multiple groups, the Kruskal-Wallis H test was employed, while pairwise comparisons were conducted using the Mann-Whitney U test. For categorical variables (expressed as ratios), chi-square tests or Fisher’s exact tests were used. A two-tailed p-value < 0.05 indicated statistical significance. Spearman rank correlation tests, z-score normalization, LR model output (displaying feature coefficients), LightGBM model feature importance output, and LASSO regression analysis were performed using Python software (version 3.7.17; http://www.python.org). ROC curves and clinical decision curves were plotted accordingly. The evaluation of the models involved AUC values, accuracy, sensitivity, specificity, PPV, NPV, and DCA, which were implemented using Python software.

## Results

### Patient characteristics

This study involved 417 patients of breast cancer, all female, from two research centers. One center contributed 274 patients to the training cohort and 68 patients to the test cohort, while the other center provided 75 patients for the Test1 cohort. Disparities were observed among the creatine kinase isoenzyme, α-hydroxybutyrate dehydrogenase, indirect bilirubin, globulin, albumin-globulin ratio, blood bicarbonate concentration, total bile acids, Na, Cl, Mg, and the maximum diameter of breast cancer lesions on ultrasound among the three cohorts (**Table 1**). Pairwise comparisons revealed differences between the Training cohort and the Test1 cohort for most markers, as well as between the Test cohort and the Test1 cohort (**Supplementary Table 1**), suggesting that the data indeed originated from two research centers, with the Training cohort and Test cohort coming from the same center. Patient ultrasound, pathological, and clinical blood marker characteristics are summarized in **Table 1**.

### Feature selection

**Figure 2:**
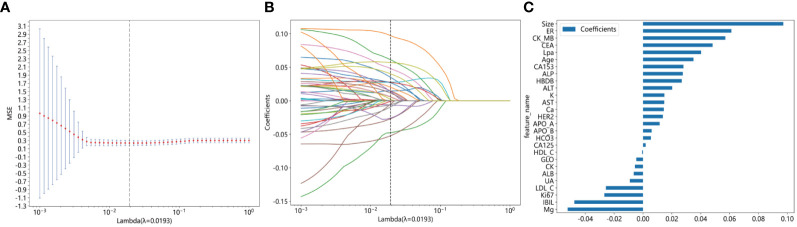
Illustrating the feature selection process using the least absolute shrinkage and selection operator (LASSO) regression model involves several crucial steps. **(A)** The horizontal axis represents different λ values, while the vertical axis represents the corresponding average mean squared error (MSE). Through 10-fold cross-validation, we calculated the average MSE for each λ value. Then, we identified the λ value corresponding to the minimum MSE, marked by a vertical dashed line on the chart. The optimal λ value is 0.0193; **(B)** The horizontal axis represents different λ values, indicating varying regularization strengths. A higher λ value indicates stronger regularization. The vertical axis represents the coefficients of each feature. When λ is small, regularization is weak, and the model may include more features, with many feature coefficients being non-zero. As λ increases, regularization strength increases, causing many feature coefficients to gradually shrink or even become zero. This is because LASSO regression tends to shrink unimportant feature coefficients to zero to simplify the model. Each curve represents the trajectory of a feature coefficient as λ changes. As λ increases, feature coefficients gradually decrease to zero. Features that decrease to zero earliest are typically those with smaller impacts on the target variable (metastatic breast cancer). Features that still maintain non-zero coefficients at the optimal λ value (indicated by the dashed line) are usually those with larger impacts on the target variable. **(C)** This figure displays the features selected by Lasso regression, with the vertical axis showing the final selected features and their corresponding coefficient values (as indicated by the horizontal axis).

The feature data were normalized and one of the features with a Spearman correlation coefficient > 0.9 was retained. Dimensionality reduction was conducted by eliminating features with zero coefficients through LASSO regression. The optimal λ value (0.0193) was determined based on the minimum Mean Squared Error (**Figure 2A**), and a Lasso regression model was fitted using the optimal λ value (**Figure 2B**). After feature dimensionality reduction, 27 features were finally selected (**Figure 2C**). Each of these features was then used independently as input for subsequent model building.

### Construction and validation of LR and LightGBM models

**Figure 3:**
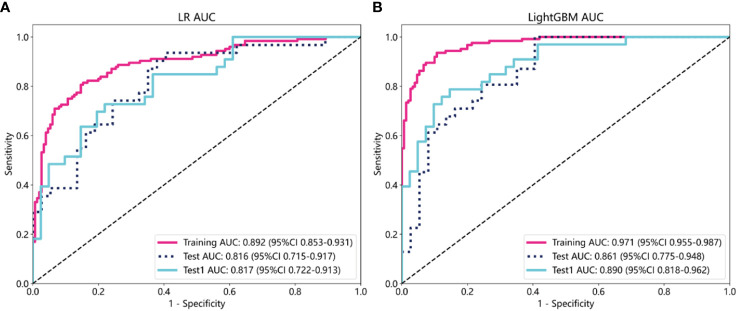
The evaluation of the Receiver Operating Characteristic curves for both the Logistic Regression **(A)** and LightGBM **(B)** models was conducted across three different datasets: the training cohort, the test cohort, and an additional independent test cohort (test1). This comprehensive evaluation allows for a thorough comparison of model performance and generalizability.

**Figure 4:**
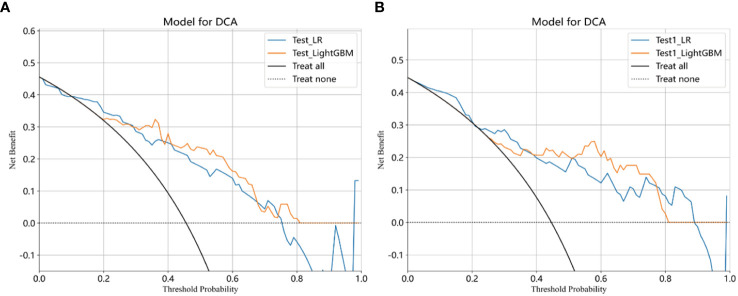
Clinical decision curves analysis (DCA) for the LR and LightGBM models constructed in the test **(A)**, and test1 **(B)** cohorts were demonstrated. Treat-All: Treating all cases as if they have metastatic breast cancer, regardless of whether the model predicts metastatic or non-metastatic stages; Treat-None: Treating all cases as if they do not have metastatic breast cancer, regardless of whether the model predicts metastatic or non-metastatic stages; Net benefit: Evaluate the practical utility of a model at different decision thresholds. A higher net benefit indicates that the model’s predictions have greater value for clinical decision-making at that threshold. Through DCA, net benefit helps determine whether the model outperforms the simple “Treat-All” or “Treat-None” strategies at different thresholds. If the model’s net benefit at a given threshold exceeds that of the “Treat-All” and “Treat-None” strategies, it suggests that using the model’s predictions is more beneficial than either extreme strategy at that threshold.

The selected features were used to construct LR, LightGBM, GBoost, and AdaBoost models, with performance parameters shown in **Table 2**. The AUC values for the LR and LightGBM models in external validation were relatively high, with the ROC curve results displayed in **Figures 3A** and **B**, respectively. The ROC for the LR model in the training, test, and Test1 cohorts was 0.892 (95% CI 0.853–0.931), 0.816 (95% CI 0.715–0.917), and 0.817 (95% CI 0.722–0.913), respectively. For the LightGBM model, the ROC was 0.971 (95% CI 0.955–0.987), 0.861 (95% CI 0.775–0.948), and 0.890 (95% CI 0.818–0.962) in the training, test, and Test1 cohorts, respectively. Other performance parameters are presented in **Table 2**. The DCA curves for both models in the training, test, and Test1 cohorts are displayed in **Supplementary Figure 1** and **Figures 4A, B**. The results indicate that the LightGBM model exhibited significantly higher net benefits at various threshold probabilities in all cohorts compared to the LR model, suggesting superior performance in identifying breast cancer with distant metastasis.

**Table 2:** Performance of models for predicting discrimination between breast cancer with distant metastasis and breast cancer without distant metastasis in training, test, and test1 cohorts.

Model	Cohort	AUC (95% CI)	Accuracy	Sensitivity	Specificity	PPV	NPV	Precision	Recall	F1	Threshold
LR	Training	0.892 (0.853 - 0.931)	0.828	0.806	0.847	0.813	0.841	0.813	0.806	0.810	0.412
	Test	0.816 (0.715 - 0.917)	0.735	0.903	0.595	0.651	0.880	0.651	0.903	0.757	0.184
	Test1	0.817 (0.853 - 0.931)	0.743	0.697	0.780	0.719	0.762	0.719	0.697	0.708	0.512
LightGBM	Training	0.971 (0.956 - 0.987)	0.909	0.927	0.893	0.878	0.937	0.878	0.927	0.902	0.440
	Test	0.861 (0.775 - 0.948)	0.765	0.968	0.595	0.667	0.957	0.667	0.968	0.789	0.366
	Test1	0.890 (0.818 - 0.962)	0.811	0.758	0.854	0.806	0.814	0.806	0.758	0.781	0.553
XGBoost	Training	1.000 (1.000 - 1.000)	0.996	0.992	1.000	1.000	0.993	1.000	0.992	0.996	0.628
	Test	0.846 (0.749 - 0.942)	0.794	0.677	0.892	0.840	0.767	0.840	0.677	0.750	0.579
	Test1	0.776 (0.668 - 0.885)	0.730	0.818	0.659	0.659	0.818	0.659	0.818	0.730	0.624
AdaBoost	Training	0.914 (0.883 - 0.944)	0.818	0.758	0.867	0.825	0.812	0.825	0.758	0.790	0.501
	Test	0.828 (0.727 - 0.929)	0.765	0.839	0.703	0.703	0.839	0.703	0.839	0.765	0.452
	Test1	0.759 (0.647 - 0.872)	0.703	0.636	0.756	0.677	0.721	0.677	0.636	0.656	0.538

### Model feature analysis

**Figure 5:**
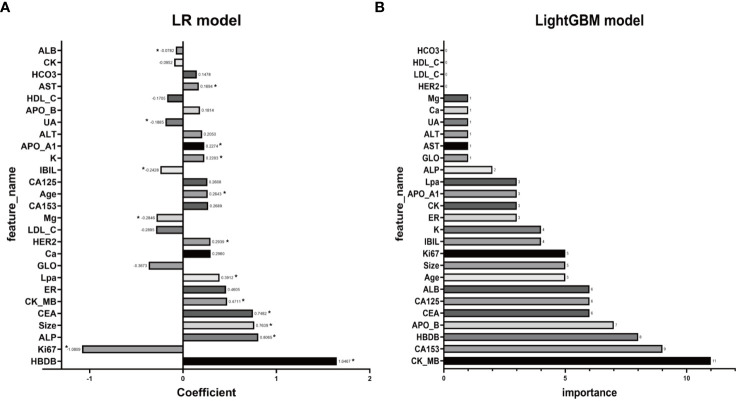
Illustrating the feature analysis aimed at identifying key features contributing to the prediction of distant metastasis, both LR **(A)** and LightGBM **(B)** models are scrutinized. In panel **(A)**, coefficients with corresponding p-values less than 0.05 will be marked with an asterisk (*) beside the coefficients.

To identify key features contributing to the prediction of distant metastasis in LR and LightGBM models, feature analysis was conducted. The results are shown in **Figures 5A, B**. In the LR model, the top 5 features with relatively significant impact on the outcome were α-HBDH, Ki67, ALP, maximum diameter of lesions on ultrasound, and CEA. In the LightGBM model, the top 5 features with relatively significant contributions were CK-MB, CA153, α-HBDH, apolipoprotein B, and CEA.

## Discussion

In this study, we employed LR and LightGBM algorithms to construct predictive models for identifying breast cancer with distant metastasis based on clinical blood markers, ultrasound examination, and breast biopsy pathology features. The LightGBM model demonstrated superior net benefits and predictive performance compared to the LR model, as evidenced by its higher AUC values in both internal and external testing datasets. These findings suggest that our models can effectively identify breast cancer patients with distant metastasis, providing clinicians with a more efficient method for early detection and intervention. This could lead to personalized treatment plans that improve patient outcomes and quality of life.

Previous studies have typically focused on assessing future metastasis risk to predict breast cancer distant metastasis. For instance, Delpech et al. and Xu et al. developed nomograms to predict bone metastasis, with C-indices ranging from 0.69 to 0.73 and 0.705 to 0.714, respectively (10, 12). Zhang et al. used MRI and ultrasound features to develop a prognostic nomogram, achieving C-indices of 0.882 and 0.812 (15). Wang et al. utilized gene expression profiles for a nomogram predicting lung metastasis risk, with C-indices of 0.862 and 0.772 (13). Additionally, Shidi Miao et al. constructed a nomogram model using CT image features of muscles and clinicopathological features, achieving C-indices of 0.983 and 0.948 in the training and test cohorts, respectively, although it was not externally validated (18). Besides these nomogram models, Li et al. used the SEER database (2010–2019) and the XGBoost algorithm to construct a model predicting survival rates in breast cancer patients with brain metastasis (AUC around 0.8), with external validation using their center’s data (AUC around 0.7) (16).

However, fewer studies have focused on diagnostic prediction models for patients with existing distant metastasis. Li et al. used radiomic features from magnetic resonance imaging (MRI) alone or combined with clinicopathological features for prediction, achieving AUC values of 0.744 and 0.763, respectively (19); however, the study did not specify the machine learning algorithms used. Huang et al. predicted bone metastasis in invasive ductal carcinoma using the SEER database, achieving an AUC of 0.907 (11). Ma et al. developed a fusion model combining clinicopathological and MRI features, achieving AUC values of 0.870 and 0.822, respectively (14). Besides the aforementioned nomogram models, other algorithms have shown performance in predicting breast cancer. For example, Zhao et al. used four machine learning algorithms to predict the risk of breast cancer distant metastasis, with XGBoost performing best (AUC of 0.907 in the training set and 0.754 in the validation set) (20). Burak Yagin et al. constructed a model predicting breast cancer distant metastasis using the genomic data of 98 breast cancer cases, with the LightGBM model performing best (21).

This study is also based on XGBoost and LightGBM and uses clinical blood markers indicative of cardiac, hepatic, and renal function, combined with ultrasound and other clinicopathological features, to construct models validated across different centers. In our external data validation, the LightGBM model performed better, achieving an AUC of 0.890.

CK-MB was identified as one of the most important features in the LightGBM model prediction. As a creatine kinase isoenzyme, CK-MB exists mainly in the myocardium and skeletal muscles (22). Previous studies have found that the ratio of CK-MB to total CK is significantly higher in advanced malignant tumor patients compared to early-stage ones (23), suggesting an association between CK-MB and cancer progression stages. Moreover, serum CK-MB activity is significantly elevated in metastatic tumor patients compared to those with primary tumors (22). Regarding the source of elevated serum CK-MB in malignant tumor patients, studies have detected a higher proportion of CK-MB in tumor tissues of lung cancer patients, implying that the increased plasma CK-MB may originate from tumor tissues rather than myocardium and skeletal muscles (24). In our study, CK-MB played a crucial role as one of the key features in the model prediction, suggesting its importance in predicting breast cancer with distant metastasis. However, further research is needed to explore why CK-MB elevation occurs in breast cancer with distant metastasis and whether elevated CK-MB originates from tumors or other sources. α-HBDH, as an LDH isoenzyme, is significantly elevated in the serum of some malignant tumor patients and is associated with the prognosis of malignant tumors (25–27). The combined application of α-HBDH, CEA, and CA125 in the early diagnosis of breast cancer has been found to be valuable (28). CA153 is a common tumor marker with predictive ability for breast cancer distant metastasis (29). In our study, CA153 was also one of the important features in model construction.

This study has some limitations. Firstly, we only included common types of distant metastases of breast cancer, such as bone, liver, and lung metastases. This means that we did not consider other types of distant metastases, such as brain metastases and post-treatment breast cancer distant metastases. The prognosis of post-treatment metastatic breast cancer may be worse because treatment may lead to the reselection of tumor molecules, making them more invasive (30). Secondly, although our data came from two different medical centers, they were both located in the same region. Therefore, our dataset may lack sufficient representativeness and requires validation across broader geographic areas, even across multiple centers internationally. Finally, due to potential differences among different healthcare institutions or equipment, the performance of our model may vary in different environments. Therefore, our model may require more validation datasets to ensure its applicability and reliability in different clinical settings.

In conclusion, this study successfully developed and validated LR and LightGBM machine learning models based on clinical blood markers, ultrasound data, and biopsy pathology features to predict distant metastasis in breast cancer patients. Particularly, the LightGBM model exhibited higher accuracy and potential clinical application value in predicting and identifying breast cancer with distant metastasis. These tools are expected to elevate the level of clinical decision-making and prognosis assessment, potentially reducing the need for expensive or invasive imaging techniques. This study highlights the prospects of using readily available clinical blood markers and cost-effective ultrasound data for developing artificial intelligence predictive tools.

In conclusion, our study successfully developed and validated LR and LightGBM models using clinical blood markers, ultrasound data, and biopsy pathology features to predict distant metastasis in breast cancer patients. The LightGBM model, in particular, demonstrated higher accuracy and potential clinical utility. These models could enhance clinical decision-making and prognosis assessment, reducing reliance on expensive or invasive imaging techniques. Our findings underscore the potential of integrating readily available clinical data and machine learning for early and accurate prediction of breast cancer metastasis.

## Data availability statement

The original contributions presented in the study are included in the article/**Supplementary Material**. Further inquiries can be directed to the corresponding author.

## Ethics statement

This study has obtained ethical approval from the Medical Ethics Committee of the First Affiliated Hospital of Guangxi Medical University (Reference Number: 2023-E749–01) and the Medical Ethics Committee of Guangxi Medical University Tumor Hospital (Reference Number: KY2023868). Due to the retrospective nature of the study, the requirement for informed consent has been waived by the Medical Ethics Committee of the First Affiliated Hospital of Guangxi Medical University and the Medical Ethics Committee of Guangxi Medical University Tumor Hospital.

## Author contributions

WZ: Writing – original draft, Visualization, Validation, Software, Resources, Investigation, Formal analysis, Data curation, Conceptualization. YT: Writing – original draft, Software, Resources, Investigation, Formal analysis, Conceptualization. ZH: Writing – original draft, Visualization, Data curation. QT: Writing – original draft, Validation, Data curation. YZ: Writing – original draft, Validation, Data curation. BC: Writing – original draft, Validation, Data curation. CW: Writing – review & editing, Supervision, Project administration, Methodology, Funding acquisition.

## Supplementary material

Supplementary Figure 1 Clinical decision curves analysis (DCA) for the LR and LightGBM models constructed in the training cohort were demonstrated.

Supplementary Table 1 The P-values for pairwise comparisons of baseline characteristics among the Training cohort, Test cohort, and Test1 cohort. For detailed explanations of the abbreviations for the variables, please refer to .
