# Supplementary figures and images for: Development and validation of AI models using LR and LightGBM for predicting distant metastasis in breast cancer: a dual-center study
Source: Front Oncol. 2024 Jun 14;14:1409273. doi: 10.3389/fonc.2024.1409273 (PMC11211559; doi:10.3389/fonc.2024.1409273)

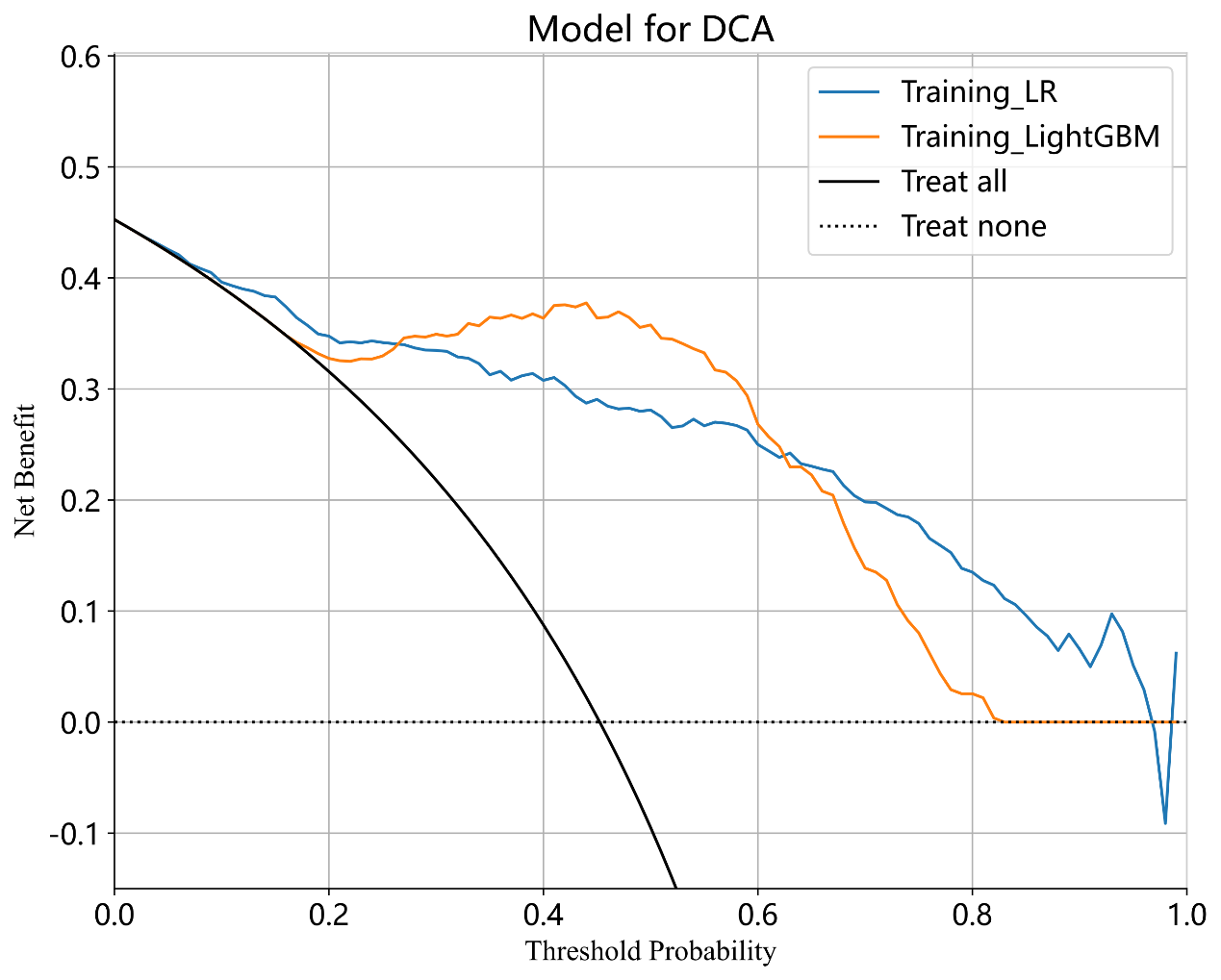

Supplement: Supplementary Figure 1 — Clinical decision curves analysis (DCA) for the LR and LightGBM models constructed in the training cohort were demonstrated. [file Image_1.tif]
