# Supplementary material for: Development and validation of AI models using LR and LightGBM for predicting distant metastasis in breast cancer: a dual-center study
Source: Front Oncol. 2024 Jun 14;14:1409273. doi: 10.3389/fonc.2024.1409273 (PMC11211559; doi:10.3389/fonc.2024.1409273)
Supplement: Supplementary Table 1 — The P-values for pairwise comparisons of baseline characteristics among the Training cohort, Test cohort, and Test1 cohort. For detailed explanations of the abbreviations for the variables, please refer to . [file Table_1.docx]

Supplementary Table 1 The P-values for pairwise comparisons of baseline characteristics among the Training cohort, Test cohort, and Test1 cohort.

| Characteristics | Training cohort VS Test cohort | Test cohort VS Test1 cohort | Training cohort VS Test1 cohort |
| --- | --- | --- | --- |
| Maximum tumor diameter by ultrasound (cm) | 0.381 | 0.015 | <0.001 |
| IBIL (μ mol/L) | 0.831 | 0.006 | 0.002 |
| GLO (g/L) | 0.731 | 0.002 | <0.001 |
| A_G (Ratio) | 0.806 | 0.001 | <0.001 |
| Na (mmol/L) | 0.242 | <0.001 | <0.001 |
| TBA (μ mol/L) | 0.003 | <0.001 | 0.017 |
| Cl (mmol/L) | 0.945 | <0.001 | <0.001 |
| Mg (mmol/L) | 0.216 | <0.001 | <0.001 |
| HCO3 (mmol/L) | 0.332 | 0.015 | 0.039 |
| CK_MB (U/L) | 0.997 | 0.002 | <0.001 |
| HBDB (U/L) | 0.736 | 0.068 | 0.006 |

For detailed explanations of the abbreviations for the variables, please refer to Table 1.
